# Ammonium 1-hydr­oxy-2-naphthoate

**DOI:** 10.1107/S1600536808009082

**Published:** 2008-04-10

**Authors:** Ye Bi, Cheng-Li Han

**Affiliations:** a College of Chemistry and Chemical Engineering, Qiqihar University, Qiqihar 161006, People’s Republic of China

## Abstract

The title compound, NH_4_^+^·C_11_H_7_O_3_^−^, was obtained by slow evaporation of a 30% ammonia solution of 1-hydr­oxy-2-naphthoic acid. The crystal structure is stabilized by inter­molecular N—H⋯O hydrogen bonds, forming layers parallel to the *bc* plane.

## Related literature

For related literature, see: Kickelbick & Schubert (1999 ▶); Ohki *et al.* (1986 ▶); Song *et al.* (2008 ▶).
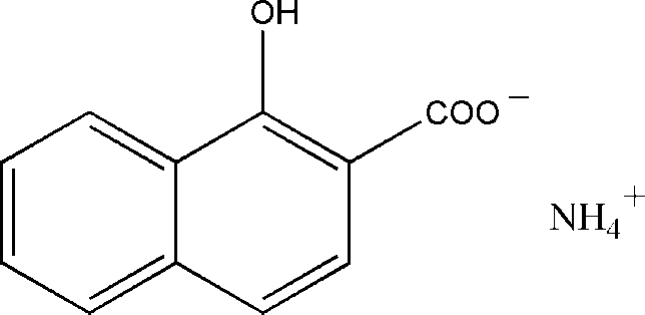


## Experimental

### 

#### Crystal data

NH_4_^+^·C_11_H_7_O_3_^−^
*M* *_r_* = 205.21
Monoclinic, 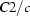

*a* = 30.883 (5) Å
*b* = 3.880 (1) Å
*c* = 15.777 (3) Å
β = 95.567 (2)°
*V* = 1881.6 (7) Å^3^
*Z* = 8
Mo *K*α radiation
μ = 0.11 mm^−1^
*T* = 298 (2) K
0.23 × 0.23 × 0.20 mm

#### Data collection

Bruker SMART CCD area-detector diffractometer
Absorption correction: multi-scan (*SADABS*; Sheldrick, 1996 ▶) *T*_min_ = 0.976, *T*_max_ = 0.979
6728 measured reflections
1915 independent reflections
1351 reflections with *I* > 2σ(*I*)
*R*_int_ = 0.040

#### Refinement

*R*[*F* ^2^ > 2σ(*F* ^2^)] = 0.080
*wR*(*F* ^2^) = 0.226
*S* = 1.04
1915 reflections
149 parameters
10 restraints
H atoms treated by a mixture of independent and constrained refinement
Δρ_max_ = 0.55 e Å^−3^
Δρ_min_ = −0.24 e Å^−3^

### 

Data collection: *SMART* (Bruker, 1998 ▶); cell refinement: *SAINT* (Bruker, 1998 ▶); data reduction: *SAINT*; program(s) used to solve structure: *SHELXS97* (Sheldrick, 2008 ▶); program(s) used to refine structure: *SHELXL97* (Sheldrick, 2008 ▶); molecular graphics: *SHELXTL* (Sheldrick, 2008 ▶); software used to prepare material for publication: *SHELXTL*.

## Supplementary Material

Crystal structure: contains datablocks global, I. DOI: 10.1107/S1600536808009082/rz2202sup1.cif

Structure factors: contains datablocks I. DOI: 10.1107/S1600536808009082/rz2202Isup2.hkl

Additional supplementary materials:  crystallographic information; 3D view; checkCIF report

## Figures and Tables

**Table 1:** Hydrogen-bond geometry (Å, °)

*D*—H⋯*A*	*D*—H	H⋯*A*	*D*⋯*A*	*D*—H⋯*A*
O1—H1⋯O2	0.82	1.73	2.463 (3)	148
N1—H1*A*⋯O1^i^	0.89 (2)	2.07 (3)	2.920 (3)	161 (3)
N1—H1*B*⋯O2^ii^	0.89 (3)	1.88 (3)	2.756 (3)	167 (3)
N1—H1*C*⋯O3^iii^	0.89 (2)	2.04 (2)	2.789 (3)	141 (3)
N1—H1*D*⋯O3^iv^	0.88 (3)	2.08 (2)	2.821 (3)	140 (3)

Symmetry codes: (i) 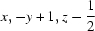; (ii) 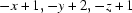; (iii) 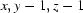; (iv) 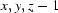.

**Fig. 1.**
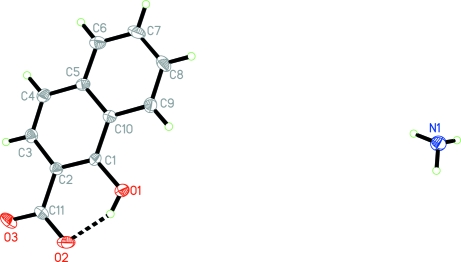
The structure of the title compound, showing 30% probability displacement ellipsoids and the atom-numbering scheme. The intramolecular hydrogen bond is shown as a dashed line.

**Fig. 2.**
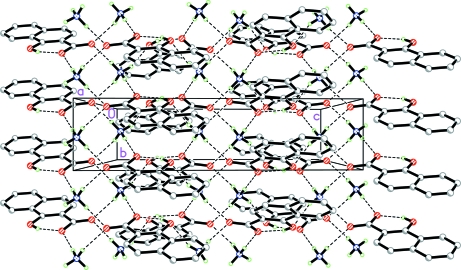
A perspective view of crystal packing of the title compound, viewed along the a axis. Hydrogen bonds are shown as dashed lines.

## supplementary crystallographic information

### Comment

1-Hydroxynaphthalene-2-carboxylic acid is a widely used ligand for the synthesis of metal complexes (Kickelbick & Schubert, 1999; Ohki *et al.*, 1986; Song *et al.*, 2008). We report herein the crystal structure of the title compound, which was obtained by slow evaporation of a 30% ammonium solution of 1-hydroxynaphthalene-2-carboxylic acid in air.

The compound consists of discrete 1-hydroxynaphthalene-2-carboxylate anions and ammonium cations (Fig. 1). The anion is substantially planar with a mean deviation of 0.015 (3) Å. The crystal structure is stabilized by intermolecular N–H···O hydrogen bonds (Table 1), forming layers parallel to the *bc* plane (Fig. 2).

### Experimental

Single crystals of the title compound were obtained by slow evaporation of a 30% ammonia solution of 1-hydroxynaphthalene-2-carboxylic acid in air.

### Refinement

Ammonium H atoms were located from a difference Fourier map and refined isotropically, with N–H distances restrained to 0.90 (1) Å, H···H distances restrained to 1.43 (2) Å, and with *U*_iso_(H) values fixed at 0.08 Å^2^. All other H atoms were placed in idealized positions and constrained to ride on their parent atoms with C–H distances of 0.93 Å, O–H distance of 0.82 Å, and with *U*_iso_(H) set at 1.2*U*_eq_(C) or 1.5*U*_eq_(O).

### Crystal data

**Table:**

N_1_H_4_^+^·C_11_H_7_O_3_^–^	*F*_000_ = 864
*M**_r_* = 205.21	*D*_x_ = 1.449 Mg m^−^^3^
Monoclinic, *C*2/*c*	Mo *K*α radiation λ = 0.71073 Å
Hall symbol: -C 2yc	Cell parameters from 1379 reflections
*a* = 30.883 (5) Å	θ = 2.5–24.1º
*b* = 3.880 (1) Å	µ = 0.11 mm^−^^1^
*c* = 15.777 (3) Å	*T* = 298 (2) K
β = 95.567 (2)º	Block, colourless
*V* = 1881.6 (7) Å^3^	0.23 × 0.23 × 0.20 mm
*Z* = 8	

### Data collection

**Table:**

Bruker SMART CCD area-detector diffractometer	1915 independent reflections
Radiation source: fine-focus sealed tube	1351 reflections with *I* > 2σ(*I*)
Monochromator: graphite	*R*_int_ = 0.040
*T* = 298(2) K	θ_max_ = 27.0º
ω scans	θ_min_ = 2.6º
Absorption correction: multi-scan(SADABS; Sheldrick, 1996)	*h* = −38→38
*T*_min_ = 0.976, *T*_max_ = 0.979	*k* = −4→4
6728 measured reflections	*l* = −20→19

### Refinement

**Table:**

Refinement on *F*^2^	Secondary atom site location: difference Fourier map
Least-squares matrix: full	Hydrogen site location: inferred from neighbouring sites
*R*[*F*^2^ > 2σ(*F*^2^)] = 0.080	H atoms treated by a mixture of independent and constrained refinement
*wR*(*F*^2^) = 0.226	*w* = 1/[σ^2^(*F*_o_^2^) + (0.1461*P*)^2^ + 0.0944*P*] where *P* = (*F*_o_^2^ + 2*F*_c_^2^)/3
*S* = 1.04	(Δ/σ)_max_ < 0.001
1915 reflections	Δρ_max_ = 0.55 e Å^−^^3^
149 parameters	Δρ_min_ = −0.24 e Å^−^^3^
10 restraints	Extinction correction: none
Primary atom site location: structure-invariant direct methods	

### Special details

**Table:**

Geometry. All e.s.d.'s (except the e.s.d. in the dihedral angle between two l.s. planes) are estimated using the full covariance matrix. The cell e.s.d.'s are taken into account individually in the estimation of e.s.d.'s in distances, angles and torsion angles; correlations between e.s.d.'s in cell parameters are only used when they are defined by crystal symmetry. An approximate (isotropic) treatment of cell e.s.d.'s is used for estimating e.s.d.'s involving l.s. planes.
Refinement. Refinement of *F*^2^ against ALL reflections. The weighted *R*-factor *wR* and goodness of fit *S* are based on *F*^2^, conventional *R*-factors *R* are based on *F*, with *F* set to zero for negative *F*^2^. The threshold expression of *F*^2^ > σ(*F*^2^) is used only for calculating *R*-factors(gt) *etc*. and is not relevant to the choice of reflections for refinement. *R*-factors based on *F*^2^ are statistically about twice as large as those based on *F*, and *R*- factors based on ALL data will be even larger.

### Fractional atomic coordinates and isotropic or equivalent isotropic displacement parameters (Å^2^)

**Table:**

	*x*	*y*	*z*	*U*_iso_*/*U*_eq_	
O1	0.42976 (6)	1.0366 (6)	0.70025 (11)	0.0383 (6)	
H1	0.4496	1.0981	0.7354	0.057*	
O2	0.46982 (6)	1.1294 (6)	0.84111 (13)	0.0472 (6)	
O3	0.44313 (7)	0.9570 (6)	0.95912 (12)	0.0486 (7)	
N1	0.46529 (8)	0.4511 (7)	0.08424 (16)	0.0414 (7)	
C1	0.39678 (8)	0.9092 (6)	0.74083 (16)	0.0259 (6)	
C2	0.39995 (8)	0.8798 (7)	0.82806 (16)	0.0281 (6)	
C3	0.36469 (8)	0.7397 (7)	0.86694 (16)	0.0322 (6)	
H3	0.3666	0.7214	0.9260	0.039*	
C4	0.32803 (9)	0.6309 (8)	0.82012 (17)	0.0354 (7)	
H4	0.3052	0.5392	0.8474	0.042*	
C5	0.32420 (8)	0.6559 (7)	0.73053 (17)	0.0291 (6)	
C6	0.28703 (9)	0.5425 (7)	0.67931 (19)	0.0380 (7)	
H6	0.2640	0.4471	0.7050	0.046*	
C7	0.28423 (9)	0.5700 (8)	0.5933 (2)	0.0441 (8)	
H7	0.2594	0.4931	0.5606	0.053*	
C8	0.31858 (10)	0.7139 (8)	0.55356 (18)	0.0424 (8)	
H8	0.3165	0.7321	0.4945	0.051*	
C9	0.35487 (9)	0.8268 (8)	0.60050 (17)	0.0358 (7)	
H9	0.3773	0.9242	0.5733	0.043*	
C10	0.35900 (8)	0.7985 (7)	0.68970 (16)	0.0268 (6)	
C11	0.43967 (9)	0.9947 (7)	0.88135 (17)	0.0321 (7)	
H1A	0.4533 (9)	0.347 (7)	0.1262 (14)	0.080*	
H1B	0.4891 (7)	0.565 (8)	0.1041 (18)	0.080*	
H1C	0.4720 (10)	0.291 (6)	0.0471 (16)	0.080*	
H1D	0.4467 (8)	0.597 (7)	0.0576 (18)	0.080*	

### Atomic displacement parameters (Å^2^)

**Table:**

	*U*^11^	*U*^22^	*U*^33^	*U*^12^	*U*^13^	*U*^23^
O1	0.0294 (10)	0.0542 (14)	0.0317 (10)	−0.0088 (9)	0.0045 (8)	0.0017 (9)
O2	0.0307 (10)	0.0587 (15)	0.0509 (13)	−0.0139 (9)	−0.0027 (9)	−0.0017 (11)
O3	0.0537 (13)	0.0560 (15)	0.0326 (11)	−0.0071 (10)	−0.0135 (10)	−0.0059 (10)
N1	0.0445 (14)	0.0356 (14)	0.0439 (14)	−0.0050 (11)	0.0037 (12)	−0.0057 (11)
C1	0.0240 (12)	0.0254 (13)	0.0293 (13)	0.0014 (9)	0.0070 (10)	−0.0002 (10)
C2	0.0273 (13)	0.0253 (14)	0.0310 (13)	0.0001 (10)	0.0003 (10)	−0.0035 (10)
C3	0.0371 (14)	0.0330 (15)	0.0269 (13)	−0.0017 (12)	0.0057 (11)	0.0026 (11)
C4	0.0329 (14)	0.0391 (17)	0.0352 (14)	−0.0060 (11)	0.0085 (12)	0.0052 (12)
C5	0.0246 (12)	0.0264 (14)	0.0359 (14)	0.0022 (10)	0.0010 (10)	0.0016 (11)
C6	0.0306 (14)	0.0337 (16)	0.0488 (16)	−0.0029 (11)	−0.0010 (12)	−0.0043 (13)
C7	0.0338 (15)	0.0469 (19)	0.0480 (17)	0.0026 (12)	−0.0146 (13)	−0.0136 (14)
C8	0.0440 (16)	0.0515 (19)	0.0289 (14)	0.0107 (14)	−0.0100 (12)	−0.0052 (13)
C9	0.0343 (14)	0.0436 (17)	0.0300 (14)	0.0060 (12)	0.0049 (11)	−0.0019 (12)
C10	0.0263 (12)	0.0239 (13)	0.0297 (13)	0.0024 (10)	0.0000 (10)	−0.0007 (10)
C11	0.0346 (14)	0.0271 (14)	0.0331 (14)	−0.0008 (11)	−0.0044 (11)	−0.0041 (11)

### Geometric parameters (Å, °)

**Table:**

O1—C1	1.349 (3)	C3—H3	0.9300
O1—H1	0.8200	C4—C5	1.410 (4)
O2—C11	1.287 (3)	C4—H4	0.9300
O3—C11	1.230 (3)	C5—C6	1.409 (4)
N1—H1A	0.89 (2)	C5—C10	1.418 (4)
N1—H1B	0.89 (3)	C6—C7	1.356 (4)
N1—H1C	0.89 (2)	C6—H6	0.9300
N1—H1D	0.88 (3)	C7—C8	1.400 (4)
C1—C2	1.375 (4)	C7—H7	0.9300
C1—C10	1.419 (3)	C8—C9	1.355 (4)
C2—C3	1.410 (3)	C8—H8	0.9300
C2—C11	1.487 (3)	C9—C10	1.405 (4)
C3—C4	1.358 (4)	C9—H9	0.9300
			
C1—O1—H1	109.5	C6—C5—C10	118.2 (3)
H1A—N1—H1B	110.8 (19)	C4—C5—C10	119.3 (2)
H1A—N1—H1C	108 (2)	C7—C6—C5	121.3 (3)
H1B—N1—H1C	110.0 (19)	C7—C6—H6	119.4
H1A—N1—H1D	110 (2)	C5—C6—H6	119.4
H1B—N1—H1D	109 (2)	C6—C7—C8	120.1 (3)
H1C—N1—H1D	108.3 (19)	C6—C7—H7	119.9
O1—C1—C2	121.5 (2)	C8—C7—H7	119.9
O1—C1—C10	117.3 (2)	C9—C8—C7	120.5 (3)
C2—C1—C10	121.2 (2)	C9—C8—H8	119.8
C1—C2—C3	119.0 (2)	C7—C8—H8	119.8
C1—C2—C11	121.1 (2)	C8—C9—C10	120.8 (3)
C3—C2—C11	120.0 (2)	C8—C9—H9	119.6
C4—C3—C2	121.4 (2)	C10—C9—H9	119.6
C4—C3—H3	119.3	C9—C10—C5	119.1 (2)
C2—C3—H3	119.3	C9—C10—C1	122.4 (2)
C3—C4—C5	120.5 (2)	C5—C10—C1	118.5 (2)
C3—C4—H4	119.7	O3—C11—O2	122.9 (2)
C5—C4—H4	119.7	O3—C11—C2	121.0 (3)
C6—C5—C4	122.5 (3)	O2—C11—C2	116.1 (2)

### Hydrogen-bond geometry (Å, °)

**Table:**

*D*—H···*A*	*D*—H	H···*A*	*D*···*A*	*D*—H···*A*
O1—H1···O2	0.82	1.73	2.463 (3)	148
N1—H1A···O1^i^	0.89 (2)	2.07 (3)	2.920 (3)	161 (3)
N1—H1B···O2^ii^	0.89 (3)	1.88 (3)	2.756 (3)	167 (3)
N1—H1C···O3^iii^	0.89 (2)	2.04 (2)	2.789 (3)	141 (3)
N1—H1D···O3^iv^	0.88 (3)	2.08 (2)	2.821 (3)	140 (3)

Symmetry codes: (i) *x*, −*y*+1, *z*−1/2; (ii) −*x*+1, −*y*+2, −*z*+1; (iii) *x*, *y*−1, *z*−1; (iv) *x*, *y*, *z*−1.
